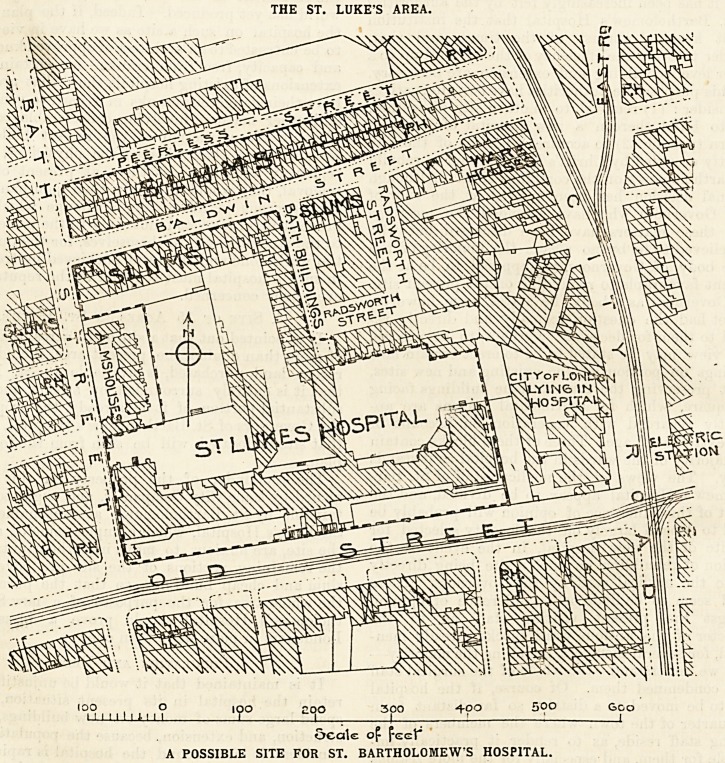# The Question of Hospital Sites in London

**Published:** 1903-01-10

**Authors:** 


### HOSPITAL ADMINISTRATION.

### CONSTRUCTION AND ECONOMICS.

### THE QUESTION OF HOSPITAL SITES IN LONDON.

We always welcome evidence of interest in the hospitals on the part of our daily contemporaries. The *Daily Mail* has devoted considerable space of late to the position of Londoners *vis-a-vis* with the hospital accommodation provided at present on existing sites. We have been consulted on this question, and in consequence have taken some pains to ascertain the facts so as to deal with it in all its aspects. It has been necessary to devote some considerable time to the investigation, for, unlike some modern daily newspapers The Hospital has, in the first instance, to verify its facts before inflicting them on its readers. It has been urged that the articles in the *Daily Mail* emanate—as most big things are now said to emanate—from the other side of the Atlantic; that behind this agitation on the question of hospital sites lies the American millionaire or his representatives in search of the best positions in the leading thoroughfares for the purpose of exploiting enterprises of various kinds. We prefer to credit the *Daily Mail* with a desire to help London and its hospitals, and this view is confirmed by one statement at least which it makes in regard to the Westminster Hospital. It states in effect that the site of the Westminster Hospital, which is less than an acre in extent, can be sold at once for £500,000. One result of our inquiries is to put us in a position to request the *Daily Mail* to produce its purchaser with the money, for we have reason to believe that we can then carry through the negotiations for sale to a successful issue without delay. If this challenge is accepted it will confirm our view of the action taken by our daily contemporary, for such a transaction would certainly benefit the Westminster Hospital and be a great service ultimately to Londoners.

#### On the General Question of Sites.

The question of site, so far as the London hospitals are concerned, when everything is carefully weighed and considered, must resolve itself for practical purposes, into a discussion as to whether or no it will be better to sell three or four existing sites, and to transplant the hospitals from them to larger areas in new districts, where the population is most congested and hospital accommodation is most needed? In passing, it may be observed as a practical point, that the in-patient and out-patient registers of the London hospitals, when examined, prove that patients do not necessarily, if at all, use the general or special hospital nearest to their places of residence, but, on the contrary, they seek relief at the hospital which, for some reason or other, most commends itself to their confidence. In theory the working classes in a big city like London must necessarily seek treatment at the nearest hospital, because in their case both time and money are precious. In practice we have satisfied ourselves that there is a large population using the hospitals which attaches more importance to other considerations, than those of time and money in the selection of a particular hospital. We propose to deal with the relatively few hospitals whose removal to other sites can be shown by the evidence obtainable to offer advantages to the various interests which they represent. We wish it to be clearly understood that our object is to secure an exhaustive discussion which will enable everyone interested to express his view and to have that view clearly recorded and weighed so that the ultimate decision shall be based upon provable facts. We shall welcome the co-operation of all who may feel moved to express an opinion, or who have a right from their position to offer one. For in no other way can the conclusion be reached which will most promote the welfare of the patients, the just claims of the hospitals, and at the same time best minister to the adequate maintenance of the medical schools.

#### St. Bartholomew's Hospital.

Taking the hospitals in the order of their importance we have first to deal with the case of St. Bartholomew's Hospital, Smithfield. This is the only hospital in the country which is able to meet the whole of its expenditure out of the revenue derived from its invested property and assets. As we are advised, if it is to remain on its present site, the majority of the Governors at present feel that they must appeal to the public for some hundreds of thousands of pounds, to enable them to defray the cost of the buildings it is proposed to erect on the new land recently acquired at a cost of £250,000. This circumstance alone will no doubt make the many clear-headed men amongst them hesitate, for, assuming that they were to succeed in raising in this way £300,000 for the new buildings and the structural alterations to the existing buildings, what would be the position of the hospital when so reconstructed? It is generally admitted that the plan upon which St. Bartholomew's Hospital is built renders it impracticable for structural alterations alone to convert the existing ward blocks into buildings which will be as efficient and up to date as a newly planned hospital fulfilling every modern requirement necessarily would be and ought to be. Then, too, it is undoubtedly the fact, dealing only with the question of buildings, that any scheme of reconstruction can merely be regarded as a temporary measure, so that the net result of such a policy must land St. Bartholomew's Hospital, say in 20 years' time, in a position when it would be necessary for the Governors to discard the whole of the buildings, which must then involve the sale of the existing site and the consequent sacrifice of the new buildings it is proposed to erect thereon to-day at a huge cost. We have thought it well to state this aspect first, because it demonstrates that what the Governors of St. Bartholomew's Hospital have to determine is, whether they will be content to tinker and temporise, or whether they will place to their credit for all time the undoubtedly high reputation which must be theirs, if they boldly face the position in all its bearings and determine that, for the wealthiest hospital in this country, nothing is good enough but the best.

### The Position from Within.

We have been fortunate enough in our examination of the problem before us to have the co-operation of a Fellow of the Royal College of Surgeons, who is also a distinguished graduate of London University and a Bartholomew's Hospital man. He informs us that the St. Bartholomew's view of the questions involved may be stated thus:—For many years it has been increasingly felt by the authorities of St. Bartholomew's Hospital that the institution is not keeping abreast of the times, and that, in order to bring the efficiency of the hospital up to a proper level rebuilding and extension are necessary. For this purpose the authorities have two alternatives to consider: (1) removal to another site altogether, and to build thereon a new hospital of the best modern type, or (2) to acquire a portion of the site recently occupied by Christ's Hospital and to rebuild St. Bartholomew's on the combined sites. Although no final decision has been arrived at, the view of those Governors who have attended the meetings when these matters have been discussed, but who are believed to be by no means the majority of the whole body of Governors, is supposed to be at the moment favourable to rebuilding on the present site. The Governors as a body, he states, have, however, not yet had the question formally and directly referred to them for decision. Perhaps, to be precise, their view may be stated to be to erect certain new buildings on portions of the existing and new sites, whilst preserving the four antique buildings facing the square, which for sentimental reasons are apparently regarded with veneration on account of their extreme old age. Three of these blocks contain the majority of the wards in the hospital as it stands to-day. The views of the medical staff of St. Bartholomew's Hospital appear to be divided, and the extent of this difference of opinion will probably be found to depend largely on the locality selected for the site of the new hospital, in the event of the question of rebuilding on such a site being directly put to the medical staff by the Governors. There would seem to be little or no difference of opinion amongst the present visiting staff as to the character of the antique ward blocks already mentioned, for several members, including the majority—or, as we understand—the whole of the surgical staff have condemned them. Of course, if the hospital were to be moved to a district so far distant from the quarter of the town where the members of the visiting staff reside, as to render it practically impossible for them, and especially for the more zealous of their number, to adequately discharge their duties to the patients, we agree, as all thoughtful people must agree, that the medical staff's duty in such a case would be to resist such a change. If, however, when all the facts are considered, a new site could be found, say, in St. Luke's, we are equally clear that the duty devolving upon the staff would be to support such a suggestion, because it entails the erection of an entirely new hospital in every way up to date and complying with every modern requirement, besides possessing the advantage which has become an essential matter in these days in regard to great city hospitals, that the site will be extensive enough to secure, that in a hospital containing 1,000 beds, there shall not be more than 70 beds to each acre. An area of 15 acres would afford ample space for that number of beds, and enable the hospital authorities to claim with truth and force that their new hospital, with its extensive balconies, its garden ground and open and pleasant spaces, so that an abundance of air and light must penetrate into every portion of the buildings from top to bottom. It might be made far and away the most advanced, scientifically perfect, and efficient establishment for the treatment of the sick in a great city that the world has yet produced. Indeed, if the planning of the hospital on such a site as we have in view were to be entrusted to an architect of approved knowledge and capacity, free from cranks—for certain recent extensions of existing hospitals show that architects have their dangerous cranks in regard to ventilation and other matters, involving considerable cost and ultimate reconstruction of the newest buildings—we anticipate, that whereas the physicians, or the relatively aged amongst them, at present object to removal, they would probably then join hands with the surgeons in advocating a change which must prove fruitful for good not only to the patients but to the students and to themselves, for the quality of the work done and the results achieved by treatment in such a hospital must redound to the reputation of everybody concerned.

### A Site of 15 Acres in St. Luke's.

It is pointed out that a site of 15 acres, that is one of more than double the present area, including the recent land purchased, is available in St. Luke's; that it is entirely surrounded by streets, and that a substantial portion of it is already the property of the Governors of St. Bartholomew's Hospital. Where that site is located will be seen from the following plan.

The remainder of the property on this site is owned by some City Companies, and ought to be obtainable by St. Bartholomew's at reasonable prices. St. Luke's Hospital, which occupies some 3½ acres of the site, are anxious to move into the country, and the remaining portions of the site are covered by slum and cheap buildings, so that the purchase of the whole area for the purposes of a new St. Bartholomew's Hospital should prove a blessing to Londoners in more ways than one.

### Population and Beds.

It is maintained that it would be unjustifiable to retain the hospital in its present situation, and to spend large sums of money on new buildings, reconstruction, and extension, because the population in a considerable area round the hospital is rapidly disappearing. St. Bartholomew's lies approximately in the centre of a district composed of the City of London and the boroughs of Holborn and Finsbury. The following table, containing the census figures, shows how the population of these three districts has altered in the last 40 years:—

| | Years, population in | | |
|---|---|---|---|
| | 1861. | 1881. | 1901. |
| City of London ... | 112,013 | 50,569 | 26,923 |
| Finsbury ... ... | 129,031 | 119,382 | 101,463 |
| Holborn ... ... | 94,074 | 78,668 | 59,405 |
| Totals ... | 335,118 | 248,619 | 187,791 |

The above figures show that the population of the City, in which St. Bartholomew's is situated, has diminished in the last 40 years by 76 per cent., and that in all the three districts taken together it has diminished by 44 per cent., *i.e.*, by nearly one-half. We are aware that those who oppose removal maintain that this exodus of about half of the population which St. Bartholomew's Hospital formerly ministered to is a matter of small importance, but we refrain from printing the obvious answer and figures which finally dispose of this view, because we do not suppose that on examination anyone will be found to continuously put it forward, as a sound argument for remaining on the existing site. Turning next to the number of beds required in the new hospital the census returns show that the population which St. Bartholomew's would minister to on the St. Luke's site is upwards of a million, for it occupies approximately the centre of Finsbury and Shoreditch and would be called upon to supply hospital accommodation not only for these districts but for portions of Islington, in addition to Bethnal Green, Stepney, and the City of London. Such a population entails the provision of a hospital containing 1,000 beds, an additional reason in all the circumstances in favour of removal, having regard to the fact that the medical school attached to St. Bartholomew's is the largest and most important in the Empire. Although the population of Shoreditch and Finsbury has shown a tendency to slightly decrease rather than to increase during the last 40 years the population of the whole district just mentioned, within the same period, has increased by upwards of 220,000 souls. Without committing ourselves one way or the other we may properly point out that the St. Luke's site appears to offer so unique an opportunity for removal that it is worthy of the fullest consideration.

THE ST. LUKE'S AREA.

A POSSIBLE SITE FOR ST. BARTHOLOMEW'S HOSPITAL.

### Finance.

Of course, it is impossible for anyone but the treasurer and his advisers to state precisely what is the actual financial position and exactly how this would be affected by the policy of removal or its alternatives. In "Bart's" circles it is held, we are informed, that if the hospital is to remain where it stands, an appeal must be made to the public for £300,000. Such a step may, however, prove fatal to the reputation of St. Bartholomew's Hospital and its managers, and result in failure for the reasons already stated. The adoption of the St. Luke's site would render any such appeal wholly unnecessary. Careful inquiries have elicited the fact that St. Luke's hospital would probably be willing to hand over to the ground landlord, *i.e.*, to the Governors of St. Bartholomew's Hospital, their site, with immediate possession on payment of some £25,000. A careful estimate of the purchase price of the remainder of the 15 acres in St. Luke's shows that the total cost of the new site, including that of clearing the whole of it ready for building operations to commence would be from £250,000 to £270,000, but we will put it at £300,000 as an outside figure. There remains for consideration the cost of building and equipping a new hospital containing 1,000 beds, including a new nursing home, an administration block, and all other requirements of a modern hospital. Here we would say that we are of opinion that such a hospital should be built with a due regard to economical considerations, and that at the outside it should not be permitted to cost when completed more than from £350 to £400 per bed. It thus appears that the St. Bartholomew's Hospital can be made the most perfect hospital in the Empire, and opened for patients for an inclusive cost at the outside of some £750,000, which sum includes a margin of £50,000 for extras. *Per contra*, we have to consider what funds St. Bartholomew's would have available to meet this charge of three-quarters of a million if the existing site were disposed of. Taking the price paid by Bart's Governors to Christ's Hospital for the land recently acquired, which is not nearly as valuable as much of the existing site of Bart's, it is relatively easy to name the cash value of the property. The site, including the recent addition, covers approximately seven acres, and at the rate of £250,000 an acre should yield the sum of £1,750,000. Thus, if these figures are even approximately accurate, as we believe them to be, it would appear that removal to the St. Luke's site not only possesses the advantages presented in the above summary of our correspondent's facts, but that it involves an addition to the revenue of St. Bartholomew's Hospital of at least £30,000 a year on the most conservative estimate, which should be derived from the investment of the balance of £1,000,000 in cash, which would result to the institution from the sale of its existing site. If the Governors felt it to be good business to pay £250,000 for a little over one acre of the site they would have to dispose of in the event of removal, we take it that in their judgment, at any rate, the price they paid to Christ's Hospital is, for business purposes, reasonable enough to enable it to be used as a basis for estimating the financial effect of the sale of the whole of their existing site.

### General Considerations.

Further, and more important still, humanity demands that the Governors should pause before they commit the hospital by tinkering to ultimate discredit and ruinous outlay. For the sentiment which naturally and properly attaches to an institution which can show an unbroken record of good work through nearly nine centuries, great and inspiriting as it undoubtedly is, cannot weigh one feather's weight, if the price entailed by its exercise involves the sacrifice of, or at least serious injury to, many lives, which must necessarily be offered up should the work of the hospital be continued within the antique blocks and under the unfavourable conditions which are alone possible on the existing site. We would add to the interesting facts which our correspondent has enabled us to publish the expression of our earnest desire that this matter should be thrashed out impartially. We shall welcome any evidence for or against the views, the figures and the facts which he has collected. His work has involved the expenditure of an amount of energy and public spirit which at least entitle him to the sincere acknowledgments of everybody interested in the best welfare of St. Batholomew's Hospital.

## Figures and Tables

**Figure f1:**